# Testing Scheme Design for Grabbing Positioning and Release Mechanism in Space Microgravity Environment

**DOI:** 10.3390/s25103010

**Published:** 2025-05-10

**Authors:** Yang Zhong, Huisen Zhang, Chao Xue

**Affiliations:** School of Physics and Astronomy, Sun Yat-sen University, Zhuhai 519082, China; zhongy286@mail2.sysu.edu.cn (Y.Z.); zhanghs9@mail2.sysu.edu.cn (H.Z.)

**Keywords:** grabbing positioning and release mechanism, space testing scheme, six degrees of freedom measurement, Monte Carlo simulation

## Abstract

In the context of a space-based gravitational wave detection mission, the grabbing positioning and release mechanism (GPRM) is tasked with ensuring that the test mass (TM) is securely fixed in the appropriate configuration at the time of the satellite launch and subsequently releasing the TM in orbit at extremely low speeds across three translational and three rotational degrees of freedom. Consequently, the assessment of the GPRM functionality in a microgravity environment is a crucial step in the advancement of gravitational wave detection technology. In this paper, we present a space testing scheme for measuring the full six degrees of freedom of the test mass following its release. This was achieved through the use of a sensing system that employed spectral confocal displacement sensors and was equipped with a vacuum system, which enabled the simulation of a vacuum environment similar to that experienced in orbit. The accuracy of the testing scheme was validated by a Monte Carlo simulation test, which demonstrated that it could achieve 5 μm and 82 μrad in translational and rotational displacement measurement, respectively, and the translational and rotational velocities were found to be 0.08 μm/s and 1.4 μrad/s, respectively, over a four-second test time.

## 1. Introduction

The observation of gravitational waves has become a reality one century after Albert Einstein initially postulated their existence in 1916 based on the general theory of relativity. In 2016, the Laser Interferometer Gravitational Wave Observatory’s (LIGO’s) detection of a gravitational wave signal from the merger of two black holes marked a significant milestone in gravitational wave astrophysics [1]. However, ground-based detectors face limitations, such as the laser arm length and Earth’s pulsations, restricting their sensitivity to signals below 10 Hz. The lower-frequency mHz band holds valuable information about cosmic events, and the development of detectors capable of probing this range is a crucial area of future research. The space environment offers greater stability, and the laser interferometer arm can be extended up to $10^{6}$ km, enhancing the sensitivity of gravitational wave detectors to lower frequency bands. In 1997, the European Space Agency (ESA) and National Aeronautics and Space Administration (NASA) initiated the first space-based gravitational wave detection mission, Laser Interferometer Space Antenna (LISA), which aims to detect gravitational waves in the $10^{-4}$–1 Hz band [2,3,4]. Subsequently, projects like TianQin [5,6,7] and Taiji [8,9] have proposed similar goals and are scheduled for deployment in the 2030s.

Space-based gravitational wave detectors usually consist of three satellites in an equilateral triangular formation, with each satellite equipped with two test masses (TMs). Each TM serves as an end mirror, forming a laser interferometer arm with another mass. Laser interferometry is used to measure the changes in the distance between TMs induced by the passage of gravitational waves, thereby inferring the signal. To ensure the accuracy of the measurements, it is essential that the TM undergoes precise free-fall motion along geodesics. This is achieved through gravitational reference sensors (GRSs) that detect and control the TM’s position via the electrode housing (EH), providing a stable environment for the TM [10,11,12]. However, due to the limited control of the EH over the TM, an additional mechanism is required to prevent collisions between the EH and the TM. In order to prevent damage to the TM due to excessive impact kinetic energy during the launch phase, it is essential that the TM be firmly locked and fixed within the EH by using a caging and vent mechanism (CVM) with an approximately 1200 N preload force [4,13]. Upon reaching the designated orbit, the TM is unlocked and handed over to a grabbing positioning and release mechanism (GPRM), which is utilized to grab the TM at any attitude from any position inside the EH with a force of no more than 70 N [4,14,15,16]. Subsequently, the TM is positioned at the center of the EH before being released into free-fall. Finally, the TM is released with a velocity not exceeding 5 μm/s to allow for capture, without hitting the EH, by the electrostatic actuation force system. For space-based gravitational wave detection missions, specific initial state requirements for the TM are specified (see Table 1) to ensure there is no collision between the EH and the TM [17], highlighting the necessity for rigorous experimental testing of the GPRM’s release performance.

The group at the University of Trento has dedicated significant effort to the exploration of ground-based methods for the characterization of TM dynamics. Many scholars focused on the modeling and analysis of the release mechanism. In 2023, Tomasi et al. developed a GPRM dynamical model [18]. They used multi-body and multi-physics software and considered the friction, misalignment, and piezo actuator behavior. Trained with a large dataset, the neural network classifier could accurately identify critical configurations, helping with the GPRM design. More recently, Dalla Ricca et al. presented a 3D lumped-parameter electromechanical model of the GPRM for the LISA Pathfinder mission [19]. Using an analytical approach, they considered complex component geometries and interactions. After extensive ground testing with laser interferometers, they found that TM–mechanism terminal component impacts caused unexpected velocities. Their model accurately predicted the mechanism’s dynamics, offering insights to reduce the impact risks.

The impact dynamics of TM release is also an important research area. In 2021, Bortoluzzi et al. studied the in-flight anomalies of the LISA Pathfinder TM release mechanism, with a focus on impacts [20]. Analyzing telemetry data, like the TM’s velocity and acceleration, they found that impacts between the TM and mechanism components caused deviations. They also considered factors like friction, stiffness, and misalignment on the impact dynamics and proposed solutions like a better surface finish and control strategy optimization. Meanwhile, Zanoni and Bortoluzzi estimated the electrostatic effects on the TM’s release dynamics using the Method of Moments [21]. They modeled the electrostatic environment around the TM and calculated the electrostatic forces and capacitances. The study found that the electrostatic contribution was relatively small and not the main cause of the in-flight non-compliance. However, it still had an impact on the TM’s motion, and proper measures should be taken to minimize its effects. Then, in 2023, Vignotto et al. studied the deviation from a linear trajectory and friction-driven lateral motion in GPRM [22]. Using experiments and simulations, they found that internal boundary friction and an asymmetrical design made the plunger move diagonally when the motion direction reversed. They also studied lubrication strategies and found that proper lubrication could improve the performance. During 2024, Vignotto and Bortoluzzi analyzed TM impact dynamics from LISA Pathfinder telemetry [23]. They developed an accurate impact-detecting algorithm, calculated the coefficient of restitution, and considered the impact effects on TM’s kinetic energy and momentum, which is important for electrostatic capture. This research helped elucidate the TM–mechanism interactions and improve the release process. Recently, Shi et al. from Sun Yat-sen University developed a dynamic model for the motion asymmetry of a GPRM actuator and introduced a control methodology of the reciprocating single step motion to suppress the nonlinear trajectories of an actuator for the TianQin project [24].

In terms of improving the measurement accuracy, there have also been many research achievements. First, in 2013, Bortoluzzi et al. studied the indirect measurement of the metallic adhesion force related to elongation under dynamic and near-zero gravity conditions [25]. Using a Transferred Momentum Measurement Facility (TMMF), they measured impulses from adhered bodies’ separation and estimated the adhesion force by analyzing the TM’s displacement and velocity. This research helped elucidate the metallic adhesive behavior. Then, in 2023, Bortoluzzi and Dalla Ricca characterized the impulses produced at the rupture of adhesive bonds using a sensing body and a laser interferometer [26]. They designed a specialized experimental setup to ensure accurate measurement of the impulses. By analyzing the data, they were able to estimate the impulse intensity and duration with a high accuracy. The research also investigated the effects of various factors on the impulse measurement, such as the noise level and the damping ratio of the system. The results provided valuable insights into the adhesive behavior and helped with improving the understanding of the mechanical interactions. Simultaneously, Zou et al. from Huazhong University of Science and Technology used a compound pendulum for the TianQin project to measure the release impulse of an aluminum cubic test mass [27]. They studied how factors like the release tip material, shape, and preload force affected the impulse, providing guidelines for optimizing the release process in the TianQin mission.

These ground-based experiments verified the dynamics of the TM in several ways, while they inherently limited the measurement of the full six degrees of freedom (6-DoFs) of the TM, encompassing both translational and rotational movements. Presently, LISA Pathfinder remains the sole experimental platform capable of assessing the GPRM performance in space [3]. It has been demonstrated that LISA Pathfinder is capable of measuring the 6-DoFs of the TM using an EH apparatus and obtaining the relative acceleration noise of the TM close to the requirement for the LISA mission.

The ground-based experiments and LISA Pathfinder have provided foundational insights. This paper introduces an innovative testing scheme to evaluate the GPRM in a space microgravity environment, addressing the critical need for the precise 6-DoF measurement of TM dynamics post-release. A novel sensing system that utilizes spectral confocal displacement sensors is proposed, enabling non-contact, high-accuracy (±1μm) measurements of the TM displacements and velocities across all six degrees of freedom. The design incorporates a vacuum system to simulate orbital conditions, mitigating cold-welding effects and validating the GPRM performance under realistic space environment constraints. A methodology for deriving the 6-DoF motion from the sensor data is developed, leveraging geometric relationships and roll–pitch–yaw matrices to solve the displacement equations, achieving resolutions two orders of magnitude finer than mission requirements (5μm in translation, 82μrad in rotation). The apparatus is compatible with deployment on space stations, such as the Chinese Space Station (CSS), and integrates vibration suppression systems to counteract residual acceleration noise, ensuring reliable measurements in microgravity.

Monte Carlo simulations rigorously validated the scheme’s robustness by accounting for installation errors, machining inaccuracies, thermal fluctuations, and sensor noise, and demonstrated translational and rotational velocity uncertainties of 0.08 μm/s and 1.4 μrad/s, respectively. Unique sensor placement strategies, with three on the x+-plane, two on y+-plane, and one on z+-plane, optimized the spatial constraints and redundancy, which enabled comprehensive 6-DoF tracking even under mechanical and environmental perturbations. This study advanced prior ground-based experiments by providing full 6-DoF measurement capabilities in orbit, surpassing LISA Pathfinder’s limitations and aligning with future missions, like TianQin and Taiji. The results confirm the scheme’s superiority over existing methods by achieving sub-micron precision and validating its suitability for verifying GPRM compliance with stringent TM release criteria (Table 1). This work established a foundational framework for the in-orbit validation of precision mechanisms, which is critical for advancing space-based gravitational wave detection technologies.

In the following sections, we outline the methodology for measuring the TM 6-DoFs in Section 2, detail the apparatus structure in Section 3, present a Monte Carlo simulation analysis of the measurement uncertainty in Section 4, and conclude with a summary of our findings in Section 5.

## 2. Methodology

The TM is a cube-shaped gold–platinum alloy with precisely engineered indentations that facilitate engagement with the GPRM. The GPRM employs a two-stage release process to ensure precise control over the trajectory of the TM and to minimize transferred momentum during the final stage of detachment. In each phase of the release procedure, the contact area and the force exerted between the TM and the GPRM are successively diminished. The objective is to achieve a low-velocity release, resulting in a free-floating TM that experiences minimal external forces and can subsequently execute high-precision free-fall motion along the geodesic.

**Figure 1:**
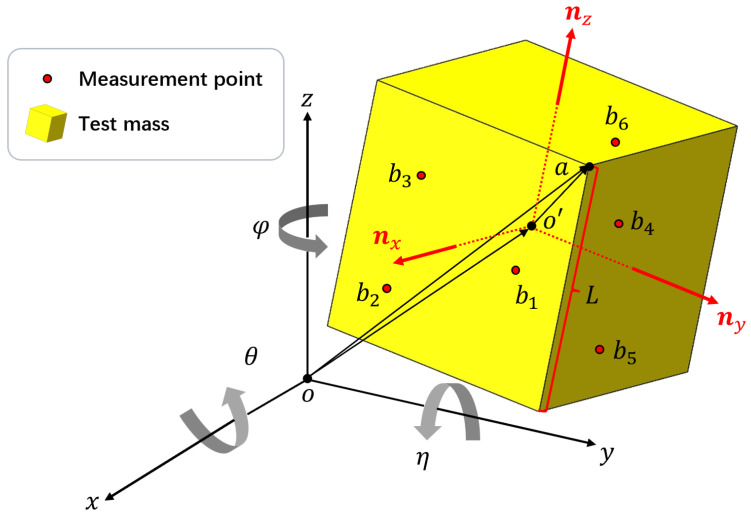
Schematic of the measuring principle. The coordinate system is fixed: the origin *o* coincides with the geometric center $o^{\prime }$ of the TM in the initial state and the three axes are perpendicular to the three planes of the TM in the initial state. The deviation between $o^{\prime }$ and *o* is the translational displacement of the TM and (θ,η,φ) represent the rotational displacement of the TM along the *x*-, *y*-, and *z*-axes.

Since the TM can be approximated as a cube, its 6-DoFs can be represented by the vector (x,y,z,φ,η,θ). A fixed coordinate system is shown in Figure 1, the origin coincides with the geometric center of the TM in its initial state, and the three axes are perpendicular to the three planes of the TM in its initial state. The coordinates (x,y,z) represent the translational DoFs of the TM center deviated from the origin, while (θ,η,φ) represent the rotational DoFs along the *x*-, *y*-, and *z*-axes. Our method involves measuring the positions of six points on the surfaces of the TM to establish a system of six equations. The 6-DoFs of the TM can then be obtained by solving this system of equations. The measurement equation is constructed as follows. Define the roll–pitch–yaw matrix:

(1) $$m\left(\varphi ,\eta ,\theta \right)=\left[\begin{array}{ccc} \cos\eta \cos\varphi & -\cos\eta \sin\varphi & \sin\eta \\ \cos\varphi \sin\eta \sin\theta +\cos\theta \sin\varphi & \cos\theta \cos\varphi -\sin\eta \sin\theta \sin\varphi & -\cos\eta \sin\theta \\ -\cos\theta \cos\varphi \sin\eta +\sin\theta \sin\varphi & \cos\varphi \sin\theta +\cos\theta \sin\eta \sin\varphi & \cos\eta \cos\theta \end{array}\right].$$

The normal vectors of the x+,y+,z+ surfaces of the TM are

(2) $$\begin{array}{cc} n_{x} & =\left(n_{xx},n_{xy},n_{xz}\right)=m\cdot \left(1,0,0\right)\\ n_{y} & =\left(n_{yx},n_{yy},n_{yz}\right)=m\cdot \left(0,1,0\right)\;.\\ n_{z} & =\left(n_{zx},n_{zy},n_{zz}\right)=m\cdot \left(0,0,1\right) \end{array}$$

Let the side length of the TM be L=2R; then, the coordinates of point *a*, which is one of the vertices of TM, can be written as

(3) $$a=\left(a_{x},a_{y},a_{z}\right)=m\cdot \left(R,R,R\right)+\left(x,y,z\right).$$

The installation of six sensors on a fixed frame that points along the coordinate axis is the first step of the process. When the TM is locked by the GPRM, the origin *o* of the coordinate system coincides with the geometric center $o^{\prime }$ of the TM, and the three axes are perpendicular to the three planes of the TM. Following the release of the TM by the GPRM, as illustrated in Figure 1, the geometric center $o^{\prime }$ of the TM will undergo movement. The measurement points correspond to the locations where the sensors are positioned on the TM surface. Given the fixed coordinates and orientation of the sensors, it follows that the measurement points will undergo movement on the surface of the TM when the TM is in motion. It is noteworthy that each measurement point possesses a single variable coordinate. For example, in Figure 1, $b_{1}$ is on the x+ surface of the TM, so this point is measured by sensor 1, which points along the *x*-axis. The coordinates of measurement point $b_{1}$ is $\left(x_{1},y_{1},z_{1}\right)=\left(R-d_{1},s_{1y},s_{1z}\right)$, where $s_{1y}$ and $s_{1z}$ are the fixed *y*- and *z*-coordinates of sensor 1 $\left(s_{1x},s_{1y},s_{1z}\right)$, and $d_{1}$ is the displacement data obtained by sensor 1, which varies with the TM movement. The coordinates of the other measurement points are determined similarly. From the installation coordinates of the six sensors $\left(s_{\alpha x},s_{\alpha y},s_{\alpha z}\right)$ and their displacement data $d_{\alpha }$, the coordinates of the six measurement points are represented as $b_{\alpha }=\left(x_{\alpha },y_{\alpha },z_{\alpha }\right),\alpha =1,2,3,4,5,6$. To ensure complete constraint of the TM’s 6-DoFs, at least one measurement point must be situated in each of the x+-, y+-, and z+-planes. In this scheme, we arrange three points on the x+-plane, two on the y+-plane, and the last one on the z+-plane, as shown in Figure 1.

Subsequently, the 6-DoFs of the TM must be derived from the six displacement measuring data due to the fact that the measurement points, denoted by $b_{\alpha }$, are located on the TM’s surface. As a result, the $ab_{\alpha }$ vectors are vertical with respect to the corresponding normal vectors $\eta_{\beta }=\left(\eta_{\beta x},\eta_{\beta y},\eta_{\beta z}\right)$ of the TM’s surfaces:

(4) $$ab_{\alpha }\cdot \eta_{\beta }=0,\left(\beta =x\;\mathrm{when}\;\alpha =1,2,3;\beta =y\;\mathrm{when}\;\alpha =4,5;\beta =z\;\mathrm{when}\;\alpha =6\right).$$

For measurement points on the x+ surface:

(5) $$n_{xx}\left(x_{\alpha }-a_{x}\right)+n_{xy}\left(y_{\alpha }-a_{y}\right)+n_{xz}\left(z_{\alpha }-a_{z}\right)=0.$$

For measurement points on the y+ surface:

(6) $$n_{yx}\left(x_{\alpha }-a_{x}\right)+n_{yy}\left(y_{\alpha }-a_{y}\right)+n_{yz}\left(z_{\alpha }-a_{z}\right)=0.$$

For measurement point on the z+ surface:

(7) $$n_{zx}\left(x_{\alpha }-a_{x}\right)+n_{zy}\left(y_{\alpha }-a_{y}\right)+n_{zz}\left(z_{\alpha }-a_{z}\right)=0.$$

The system of equations can be solved to yield six equations, and the solution to this system will provide the 6-DoF motion (x,y,z,θ,η,φ) of the TM.

The initial 6-DoF velocities of the TM can be obtained through linear fitting of the displacement data. This is predicated on the assumption that the TM is not subject to external forces and that the interference term is injected during the Monte Carlo simulation. The displacement sensor employed in this study had a sampling rate of 400 Hz, and the calculation of the 6-DoF displacements at each sampling time was facilitated by Equation (4). The 6-DoF velocities can be obtained by linear fitting of the displacement data:

(8) $$\begin{array}{cc} \; & \mathrm{linear}\;\mathrm{velocity}:\;v_{i}=it,\left(i=x,y,z\right),\\ \; & \mathrm{angular}\;\mathrm{velocity}:\;\omega_{k}=kt,\left(k=\theta ,\eta ,\varphi \right). \end{array}$$

## 3. Design of In-Orbit Testing

**Figure 2:**
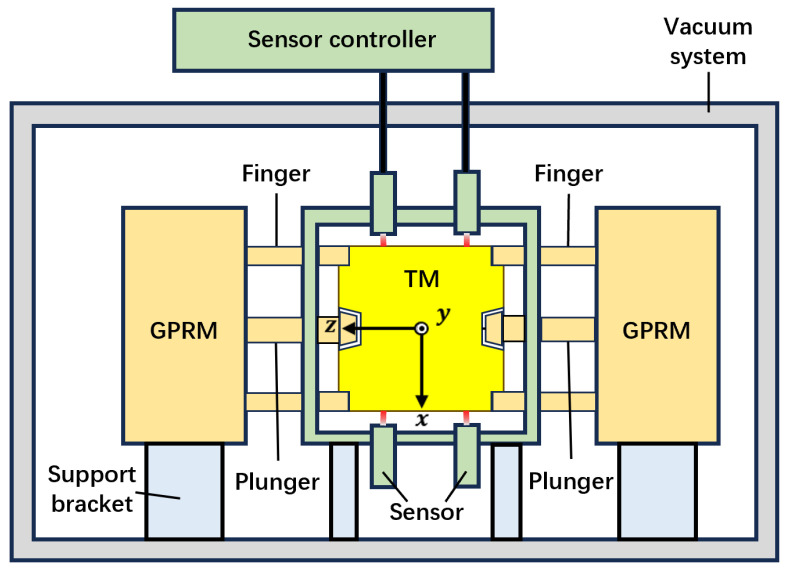
The schematic diagram of the apparatus structure. The sensing system (green) can read out the displacement data from the motion of the TM (yellow) after release; the GPRM (orange) can grab and release the TM from both sides by its fingers and plungers; the whole testing apparatus should be placed and fixed by support brackets (blue) inside a vacuum environment provided by the vacuum system (grey).

This section presents the comprehensive design of the testing apparatus, which was developed in alignment with the selected sensor and methodology. As shown in Figure 2, the apparatus structure of the testing scheme was divided into three constituent subsystems:

- Sensing system: This system acquired displacement data from the TM motion following its release and translated it into the TM’s 6-DoF format using a methodology previously described. It comprised a frame; four specific covers for mounting displacement sensors; and two sets of these sensors, each comprising six sensors.
- The GPRM and the TM: The testing objects. The GPRM maintained a firm grasp on the TM until the testing environment was prepared for its release, at which point the TM was released into a free-fall state.
- Vacuum system: The objective of this subsystem was to simulate the space-based gravitational wave detection program’s vacuum environment. In such an environment, even with minimal contact force between the TM and GPRM, notable cold-welding effects can occur, which have the potential to significantly impact the GPRM release. Consequently, the testing apparatus was housed within a vacuum chamber. The displacement sensor’s optical fibers were connected to controllers situated outside the chamber.

In the apparatus, the coordinate system in Section 2 is detailed as follows: the origin *o* ideally coincides with the geometric center of the frame, the *z*-axis runs along the GPRM’s fingers and points left, the *x*-axis runs along the support bracket and points downward perpendicular from the base, and the *y*-axis is perpendicular to the two axes above in the direction specified by the right-hand system.

### 3.1. Sensing System

To accurately measure the TM’s motion in our methodology, the chosen displacement sensor must meet the following criteria:

- Compact Size: the arrangement of the measurement points requires the displacement sensors to be mounted around at least three surfaces of the TM, especially when there is a GPRM near the z+- or z−-plane; therefore, the volume of the displacement sensor should be limited strictly.
- High accuracy: since the measurement accuracy of the linear velocity should be better than 1 μm/s, it is estimated that the measurement accuracy of the displacement sensor should be at least the same order of magnitude as 1 μm.
- Specular reflection principle: as the surface of the TM is a planar reflective plane with high flatness, the displacement sensors based on the principle of diffuse reflection to measure are banned.
- Inclined plane measurement: the TM has an initial velocity of rotation after release, and its surfaces are also rotating correspondingly, so the displacement sensor should be able to measure the displacement of the measurement point in the inclined plane.
- Non-contact operation: the displacement sensor should be a non-contact measurement type, which will not hinder the motion of the TM when installed at the working distance, and the measuring range should be able to cover the order of mm to obtain sufficient displacement data.

**Figure 3:**
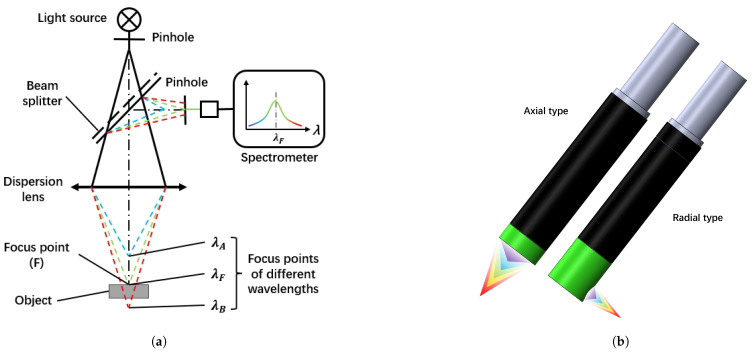
Spectral confocal displacement sensor. (**a**) The schematic diagram of the spectral confocal displacement sensor. (**b**) Two types of the spectral confocal displacement sensor.

The spectral confocal displacement sensor [28] fulfills the criteria mentioned above effectively. Figure 3a presents a schematic diagram of this sensor. The spectral confocal displacement sensor uses a broadband light source and a dispersion objective lens to generate the axial dispersion [29]. Based on the chromatic aberration principle, the TM surface’s axial position corresponds to the reflected spectrum’s foca; wavelength. Thus, the chromatic confocal system can quickly obtain the TM surface’s axial position by extracting the focal wavelength from the reflected spectrum. Furthermore, this sensor measures the displacement of points on an inclined plane without compromising accuracy. This is because the displacement measurement principle relies on the presence of reflected light rather than its intensity. As long as reflected light returns to the sensor, a displacement signal can be obtained. In addition, spectral confocal displacement sensors are available in small sizes, most of which have an accuracy higher than 1 μm.

There are two types of small-sized spectral confocal displacement sensors: axial and radial sensing (Figure 3b). For this testing scheme, the radial type is suitable for the z-plane due to the GPRM in the z-direction limiting the installation space of the sensor.

For a LISA space-based gravitational wave detection mission, a trade-off between the TM mass and the actuation noise was to be achieved. Consequently, the TM–EH separations were designed to 4.0 mm along the *x* measurement axis, 2.9 mm along the *y*-axis, and 3.5 mm along the *z*-axis, respectively [4,16,30]. The side length of the TM was set at approximately 50 mm, which constrained the TM’s motion within the frame to a maximum of 58 mm. Given the anticipated low release speed of the TM, continuous measurement of its entire motion stroke is unnecessary in GPRM testing. Reducing the required motion stroke directly decreases the test duration. Moreover, spectral confocal displacement sensors with a smaller range provide a higher measurement accuracy. The frame in our scheme was designed with a side length of 54 mm, considering the above reasons. Therefore, the working distance of the selected spectral confocal displacement sensor should be greater than 2 mm, otherwise the motion of the TM will be affected.

The design of this scheme is expected to use the spectral confocal displacement sensor of Shenzhen LightE-Technology Company, the lens model D8A15R4S30, which is divided into two types: axial and radial sensing. They have the same measurement range of ±2.12 mm, which can cover the complete motion stroke of the TM, the same maximum allowable inclination angle ±15° (a simple calculation shows that the maximum inclination angle of the TM in the frame of 54 mm is less than 5°), and the same measurement accuracy of 1 μm. The working distance of the axial type is 11.1 mm, and the radial type is 3.5 mm, both of which are more than 2 mm. The controller model they are compatible with, the E-Series, has a sampling rate of 500 Hz, allowing four sensor lenses to be connected through optical fiber at the same time.

**Figure 4:**
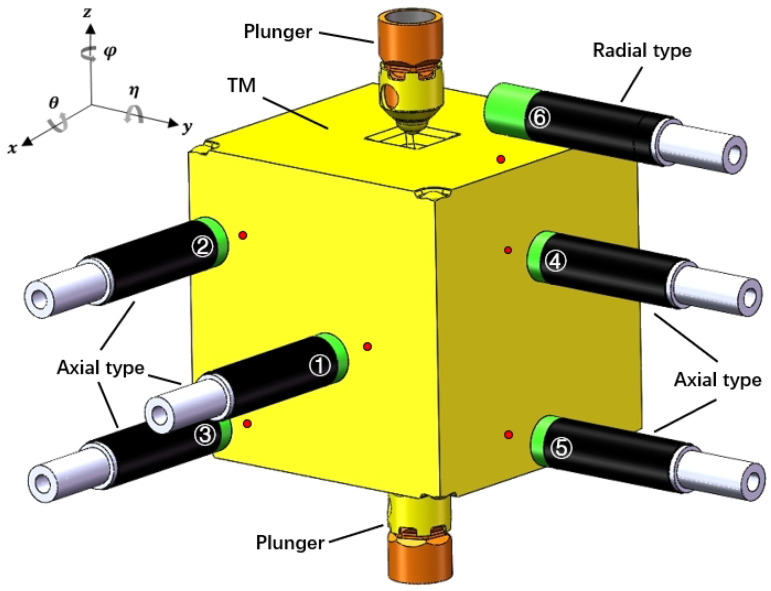
The relative positions between a set of sensors and the TM in the initial state.

As Section 2 says, we specifically designed the sensor mounts so that three sensors were pointed to the x+-plane, two pointed to the y+-plane, and one pointed to the z+-plane. Among these sensors, the sensor that pointed to the z+-plane utilized the radial type, while the remaining sensors employed the axial type, as shown in Figure 4. In detail, the installation coordinates of the six sensors (i.e., the coordinates of the center point of the optical outlet of the sensors) were designed as follows:

(9) $$\begin{array}{ccc} S_{1X} & =\left(s_{1x},s_{1y},s_{1z}\right)=\left(36.1,12.0,-1.0\right) & \mathrm{mm}\\ S_{2X} & =\left(s_{2x},s_{2y},s_{2z}\right)=\left(36.1,-12.0,14.0\right) & \mathrm{mm}\\ S_{3X} & =\left(s_{3x},s_{3y},s_{3z}\right)=\left(36.1,-12.0,-16.0\right) & \mathrm{mm}\\ S_{4Y} & =\left(s_{4x},s_{4y},s_{4z}\right)=\left(12.0,36.1,14.0\right) & \mathrm{mm}\\ S_{5Y} & =\left(s_{5x},s_{5y},s_{5z}\right)=\left(12.0,36.1,-16.0\right) & \mathrm{mm}\\ S_{6Z} & =\left(s_{6x},s_{6y},s_{6z}\right)=\left(0.0,20.0,28.5\right) & \mathrm{mm} \end{array}$$

Such installation coordinates ensured that the distance between the sensor outlet and the corresponding surface of the initial TM was equal to the working distance, and the displacement within ±2.12 mm could be measured, which effectively encompassed the entire TM motion stroke. Moreover, these coordinates prevented the sensor from measuring points on non-planar surfaces (specific indentations of the TM) during the TM motion.

The spectral confocal displacement sensors exhibited distinct noise profiles critical to the Monte Carlo simulations. Short-term variations in the sensor are characterized by two main factors. First, temporal noise, which contributes to the sensor’s ±1 μm measurement accuracy, is caused by photon shot noise and electronic noise. The manufacturer quantified this noise as a Gaussian distribution with a standard deviation of 0.3 µm at a 500 Hz sampling rate. Additionally, environmental coupling, such as high-frequency vibrations (>10 Hz) in the CSS environment, can induce transient displacements. However, this can be mitigated by the active vibration isolation system’s common-mode suppression. Residual noise (5 $\mu g/\sqrt{\mathrm{Hz}}$) contributes less than ±0.1 μm to the short-term uncertainty over 4-s tests.

Long-term drift in the sensor is mainly influenced by two aspects. Thermal drift is a significant factor, as the sensor’s low thermal coefficient and the frame expansion lead to drift when there are ±1 °C temperature fluctuations. Over a period of 4 s, this results in a systematic error of ±0.5 μm in the translational degrees of freedom. Additionally, calibration stability is crucial; the sensors’ wavelength calibration shows a drift of less than 0.1 nm/h, which is equivalent to less than a 0.1 μm drift for more than 1 month under vacuum conditions. Due to this, pre-test calibration using reference targets embedded in the frame is essential to ensure accurate measurements.

### 3.2. The GPRM and the TM and Vacuum System

Furthermore, an additional set of six sensors was incorporated into the testing scheme. The installation coordinates of the second set of sensors were used to rotate the coordinates of the first set (Equation (9)) by 180° along the *z*-axis, so the second set of measurement points were distributed on x−-,y−-, and z+-planes. The purpose of this design was (1) so that these two sets of independent measurement points could be regarded as four sets of measurement points (x−y−z+; x−y+z+; x+y+z+; x+y−z+), in which the two independent sets of measurements could improve the measurement accuracy, and four sets of non-independent measurement points could be used for comparison and verification, and (2) to provide a redundant backup in the space experiment; if any sensor within the apparatus malfunctions, the other set can be utilized to complete the test.

**Figure 5:**
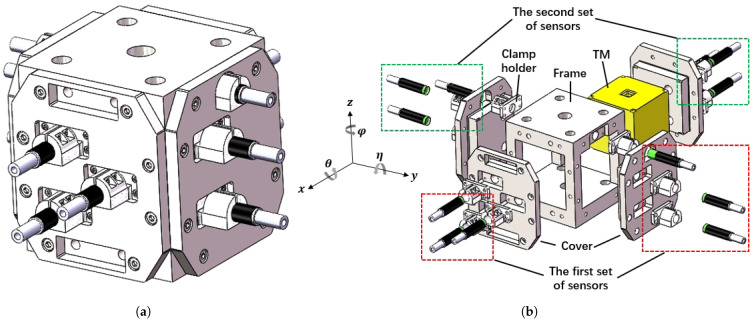
Apparatus structure of the sensing system. (**a**) Design of the sensing system. (**b**) Exploded view of the sensing system.

At last, the TM was confined to a frame with an inner surface side length of 54 mm. Considering loading the TM into the frame, it should feature an open design, as shown in Figure 5. The sensor needs to be fixed to the clamp holder, which is then mounted to the frame at the designated position.

**Figure 6:**
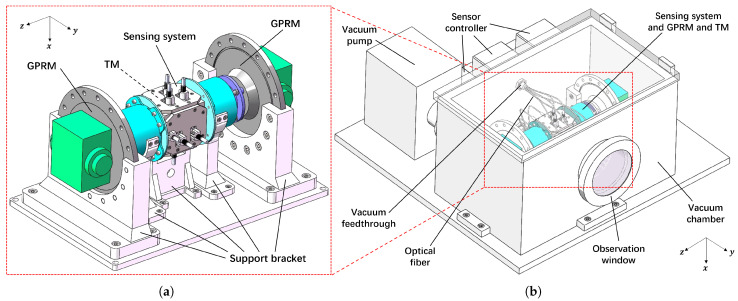
Structure diagram of the testing apparatus. (**a**) The installation of the sensing system and GPRM. (**b**) Design of the complete testing apparatus.

The sensing system and the GPRM were fixed with support brackets and mounted within the distance so that the GPRM could still grab the TM. The apparatus structure design is shown in Figure 6a.

The final configuration incorporates the integration of the sensing system and GPRM within a vacuum chamber, which resulted in the complete testing apparatus. This arrangement is illustrated in Figure 6b. The sensors were connected to their controllers through the vacuum feedthrough on the vacuum chamber by optical fibers, and the vacuum system was equipped with a vacuum pump and an observation window. With the apparatus of the testing scheme, we could test the performance of the GPRM in the space microgravity environment by obtaining the initial velocity of the TM through the methodology above and releasing the TM repeatedly to obtain a more convincing testing result.

On the space station, residual acceleration noise can cause the TM to collide with the frame after release. To mitigate this issue, the scheme was deployed within the microgravity active vibration isolation system of the China Space Station [31], whose residual gravitational acceleration values are shown in Table 2. Although the microgravity active vibration isolation system effectively prevents collisions caused by space station vibrations, its low-frequency noise (<1 Hz) remained comparable in magnitude with the initial velocity of the TM, which made it impossible to measure the initial velocity of the TM accurately. We used accelerometers for common-mode suppression to eliminate the vibration of the microgravity active vibration isolation system in the data. Due to the low accuracy of the accelerometer in the low-frequency part of the space station, we only used it to suppress the residual acceleration noise in the high-frequency part and used the MEMS Microgravity Measurement Module designed by the Huazhong University of Science and Technology [32], which suppresses residual acceleration noise in the low-frequency section. The residual acceleration noise (vibration noise) after the common-mode suppression is shown in Table 3.

The testing apparatus was designed with stringent adherence to the payload constraints of the Chinese Space Station (CSS). The total mass of the system, including the vacuum chamber, sensing system, GPRM, and support brackets did not exceed 30 kg, and thus, was well within the typical experimental payload limit for CSS microgravity experiment racks. The compact dimensions of the vacuum chamber (did not exceed 600 mm × 400 mm × 400 mm) aligned with the standard rack-mountable payload volume, which ensured compatibility with the CSS infrastructure. The vacuum system employed a space-rated turbomolecular pump, with a power consumption that generally did not exceed one hundred watts, which is compatible with the CSS’s power supply. Furthermore, the apparatus utilized the CSS’s existing microgravity active vibration isolation system without requiring structural modifications, as the support brackets were designed to interface directly with active vibration isolation system mounting points. These design choices ensured that the testing scheme met the CSS’s technical and operational requirements, which confirmed its feasibility for actual deployment.

## 4. Results

To assess the accuracy of the testing scheme, a Monte Carlo simulation test was performed using a simulation environment that included the following:

- Installation error: The error in the installation accuracy of the displacement sensors, including the position and inclination installation error. These would cause the values in Equation (9) to deviate from the expected values.
- Machining accuracy error: Machining accuracy errors of the TM and the frame. This causes the edge lengths of TM and frames to deviate from the expected values.
- Environment temperature fluctuation: due to the thermal expansion effect, the temperature fluctuation of the environment will deviate the edge lengths of the TM and frames to deviate from the expected values.
- Vibration noise: Residual gravitational acceleration noise in the space station after common-mode suppression by accelerometers. This would cause the displacement sensor’s output to mix the displacement signal of the TM with the residual vibration noise.
- Measurement accuracy: displacement measurement accuracy of the spectral confocal displacement sensor.

Their specific values are written in Table 4.

In the Monte Carlo simulations, the initial conditions were randomly chosen through a uniform distribution from the following variation ranges:

(10) $$\left|\begin{array}{c} r_{0}\\ \vartheta_{0} \end{array}\right|\leq \left[\begin{array}{c} 455\;\mu m\\ 16\;\mathrm{mrad} \end{array}\right]\;\left|\begin{array}{c} {\dot{r}}_{0}\\ {\dot{\vartheta }}_{0} \end{array}\right|\leq \left[\begin{array}{c} 23\;\;\;\mu m/s\\ 685\;\mu \mathrm{rad}/s \end{array}\right],$$

where the r is the translational displacement and the ϑ is the rotational displacement. These values were the worst release conditions experienced by LISA Pathfinder.

While the data collection ideally commences several seconds before the TM release by the GPRM to capture any anomalies during the release process, our simulation focused on evaluating the measurement apparatus’ capabilities. Consequently, the simulation initiated precisely at the moment the GPRM released the TM. To illustrate the velocity-fitting process, we present an example with specific initial velocities assigned to the TM:

(11) $$\begin{array}{c} \left(\dot{x},\dot{y},\dot{z}\right)=\left(2,-5,8\right)\;\mu m/s,\\ \left(\dot{\varphi },\dot{\eta },\dot{\theta }\right)=\left(80,-40,150\right)\;\mu \mathrm{rad}/s, \end{array}$$

**Figure 7:**
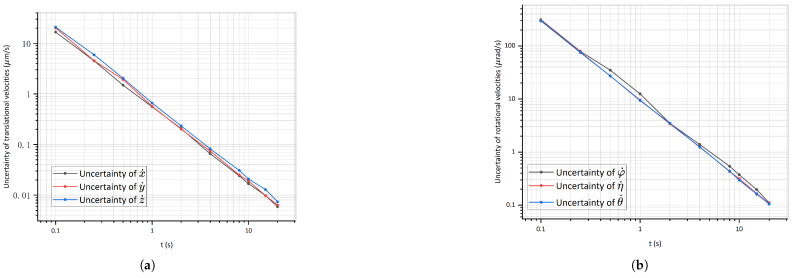
Uncertainties in the 6-DoF velocities over time. (**a**) shows the uncertainties in the translational velocities. (**b**) shows the uncertainties in the rotational velocities.

To determine the optimal test time for achieving the desired velocity measurement accuracy, we investigated the impact of different test times on the 6-DoF velocity deviation from the exact value (Figure 7). Over a quarter of a second test time, the accuracy of the translational and rotational velocities reached the order of the initial velocity. For accurate and stable measurements, a test time of 4 s was suggested, whose accuracy of the translational and rotational velocities was two orders of magnitude smaller than the initial velocity required in Table 1.

**Figure 8:**
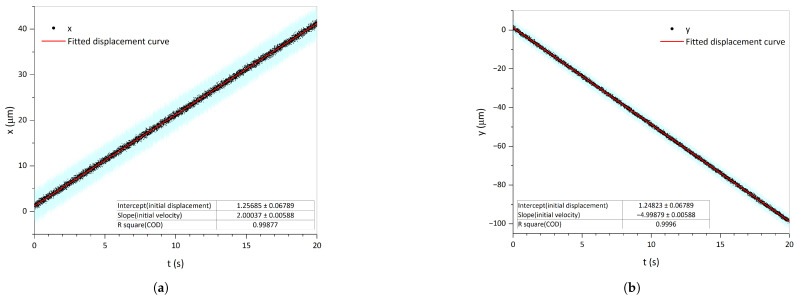

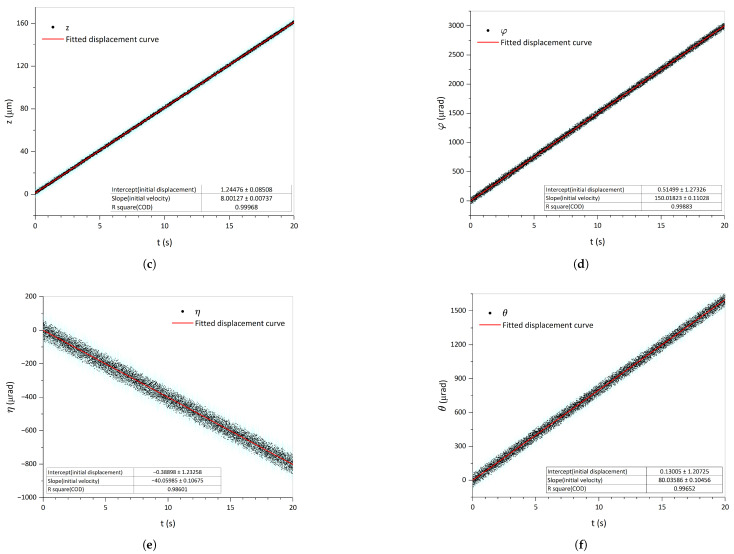
Velocity-fitting example. Each line illustrates the evolution of the noisy 6-DoF displacement over time, with the blue error bars at each black point being the maximum error in different environments derived from Monte Carlo simulations. The initial 6-DoF velocities of the TM could be fitted from this, and the solid red line is the fitted displacement curve. (**a**) Linear velocity along the *x*-axis. (**b**) Linear velocity along the *y*-axis. (**c**) Linear velocity along the *z*-axis. (**d**) Angular velocity around the *z*-axis. (**e**) Angular velocity around the *y*-axis. (**f**) Angular velocity around the *x*-axis.

Figure 8 presents the evolution of the noisy 6-DoF displacement over time, with each line representing a different degree of freedom. The error bars at each point depict the maximum error across different environments derived from Monte Carlo simulations. The initial 6-DoF velocities of the TM could be fitted from this, and the solid red line is the fitted displacement curve. Within a 20-s test period, the accuracy of the translational velocities reached the order of 0.001 μm/s, and the accuracy of the rotational velocities reached the order of 0.1 μrad/s.

The standard deviation of the 6-DoF uncertainty over the 4-s test time are shown in Table 5. The accuracy of the three translational degrees of freedom has reached the order of 1 μm, and the accuracy of the three rotational degrees of freedom has reached the order of 16 μrad. Table 6 summarizes the measurement capabilities of the scheme, noting that uncertainties for the translational z and rotational φ were slightly larger than in other directions, indicating that the *z*-axis was the non-sensitive direction. Notably, all errors fell below the required values (two orders of magnitude smaller than those specified in Table 1), confirming that the designed testing scheme effectively met the performance verification requirements for the GPRM.

The Monte Carlo simulations rigorously quantified how sensor-specific noise profiles propagated into 6-DoF uncertainties, which validated the scheme’s robustness. By addressing both short-term variations (via high sampling rates and vibration suppression) and long-term drift (through thermal design and calibration), the testing apparatus ensured reliable performance in the CSS environment. These analyses underscored the scheme’s superiority over static noise models used in ground-based experiments.

## 5. Conclusions

In order to test the performance of the GPRM in a microgravity environment, a testing scheme was developed that incorporates a vacuum system and a sensing system for measuring the initial 6-DoF state of the TM after release. This comprises two sets of 12 spectral confocal displacement sensors and their controllers, a vacuum-sealing structure, support brackets, and a specific frame. The microgravity environment provided by the space station resources and the vacuum environment provided by the vacuum system could simulate the real environment of the in-orbit TM release. According to the measurement method provided above, the 6-DoFs of the TM could be measured through the displacement data collected by these sensors, which enabled a rigorous evaluation of the performance of the GPRM.

To validate the accuracy of this testing scheme, we conducted extensive Monte Carlo simulations. In 200 simulations, regarding the 6-DoF velocities, the maximum uncertainties of the translational and rotational velocities were, respectively, 0.08 μm/s and 1.4 μrad/s over a 4-s test time; regarding the 6-DoFs, the maximum uncertainties of the translational and rotational displacements were 5 μm and 82 μrad, respectively, and the standard deviations of the translational and rotational displacement uncertainties were about 1 μm and 16 μrad, respectively. These results demonstrate that the testing scheme consistently surpassed the required accuracy by achieving uncertainties two orders of magnitude smaller than those specified in Table 1. Given the results above, this testing scheme can meet the requirements and accuracy to verify the GPRM performance and can be considered for future performance verification of the GPRM in the space microgravity environment.

Table 7 compares key quantities against ground-based experiments and the LISA Pathfinder mission. The advantages of the proposed scheme are shown as follows:

- Superior precision: Achieves higher accuracy in displacement and velocity measurements compared with the ground-based methods, which is critical for meeting the stringent TM release requirements (Table 1). The capacitive sensors of the LISA Pathfinder have a very high accuracy and are specially designed for the LISA mission. The sensor structure and circuit design are very complex.
- Environmental fidelity: unlike ground-based experiments, the vacuum system and microgravity vibration suppression (Table 3) replicate orbital conditions, addressing limitations such as air damping and seismic noise.
- Universality: the modular sensor placement and methodology are adaptable to diverse TM geometries and missions (e.g., TianQin, Taiji), whereas LISA Pathfinder’s design was mission-specific.

The proposed scheme bridges the gap between ground-based simulations and full-scale missions, like LISA Pathfinder, offering a versatile, high-precision platform for validating the GPRM performance. By outperforming prior methods in accuracy and environmental realism, it establishes a new benchmark for 6-DoF testing in space gravitational wave detection. Future work will focus on miniaturizing the apparatus for broader mission compatibility.

## Figures and Tables

**Table 1:** The requirement of the TM initial state to ensure that the EH does not collide with the TM.

State	Value	Unit
Translation	±200	μm
Rotation	±2000	μrad
Linear velocity	±5	μm/s
Angular velocity	±100	μrad/s

**Table 2:** The residual gravitational acceleration of China Space Station’s microgravity active vibration isolation system.

Frequency Band	Value	Unit
0.01–0.3 Hz	≤$5.4\times 10^{-6}$	$g_{\mathrm{RMS}}$
0.3–100 Hz	≤$1.8\times 10^{-5}$	$g_{\mathrm{RMS}}$
100–300 Hz	≤$1.8\times 10^{-3}$	$g_{\mathrm{RMS}}$

**Table 3:** The residual acceleration noise after the common-mode suppression.

Frequency Band	Value	Unit
0.01–10 Hz	±0.25	ng/$\sqrt{\mathrm{Hz}}$
10–300 Hz	±5	$\mu g/\sqrt{\mathrm{Hz}}$

**Table 4:** The noises of the simulation environment.

Quantity	Value	Unit
Position installation accuracy of the displacement sensor	±3	μm
Inclination installation accuracy of the displacement sensor	±1	mrad
Machining accuracy of the TM and the frame	±3	μm
Environment temperature fluctuations	±1	°C
Measurement accuracy of the displacement sensor	±1	μm

**Table 5:** The standard deviation of the 6-DoF uncertainty over a 4-s test time.

Quantity	Value	Unit
The standard deviation of *x* uncertainty	±1.0	μm
The standard deviation of *y* uncertainty	±1.0	μm
The standard deviation of *z* uncertainty	±1.1	μm
The standard deviation of φ uncertainty	±16.7	μrad
The standard deviation of η uncertainty	±15.7	μrad
The standard deviation of θ uncertainty	±15.8	μrad

**Table 6:** The maximum uncertainty of 6-DoFs and 6-DoF velocities.

Quantity	Value	Unit	Test Time
*x*	±4.0	μm	-
*y*	±4.7	μm	-
*z*	±5.2	μm	-
φ	±81.5	μrad	-
η	±65.8	μrad	-
θ	±66.0	μrad	-
$\left|\dot{x}\right|$	≤0.066	μm/s	≥4 s
$\left|\dot{y}\right|$	≤0.074	μm/s	≥4 s
$\left|\dot{z}\right|$	≤0.082	μm/s	≥4 s
$\left|\dot{\varphi }\right|$	≤1.4	μrad/s	≥4 s
$\left|\dot{\eta }\right|$	≤1.2	μrad/s	≥4 s
$\left|\dot{\theta }\right|$	≤1.2	μrad/s	≥4 s

**Table 7:** Comparison of the key quantities against ground-based experiments and the LISA Pathfinder mission.

Quantity	Proposed Scheme	Ground-Based Experiments [27]	LISA Pathfinder [30]
Translational accuracy	5 μm (displacement), 0.08 μm/s (velocity)	5 μm (displacement), <200 μm/s (velocity)	<2 nm (displacement)
Rotational accuracy	82 μrad (displacement), 1.4 μrad/s (velocity)	None (displacement), <1200 μrad/s (velocity)	<200 nrad (displacement)
Environment	Simulated space vacuum (10^−6^ Pa)	Atmospheric pressure (10^5^ Pa)	In-orbit vacuum (10^−6^ Pa)
Measurement DoFs	Full 6-DoFs	Limited to 1-DoF (translational)	Full 6-DoFs
Sensor type	Spectral confocal displacement sensors	Electronic autocollimator	Capacitive sensors

## Data Availability

The data that support the findings of this study are available from the corresponding author upon reasonable request.

## Abbreviations

The following abbreviations are used in this manuscript:

GRS	Gravitational reference sensor
TM	Test mass
EH	Electrode housing
GPRM	Grabbing positioning and release mechanism
CVM	Caging and vent mechanism
TMMF	Transferred momentum measurement facility
LIGO	Laser Interferometer Gravitational Wave Observatory
LISA	Laser Interferometer Space Antenna
ESA	European Space Agency
NASA	National Aeronautics and Space Administration
CSS	Chinese Space Station
DoF	Degree of freedom
